# Intrauterine Device Use and Perceptions Among Women in Tanzania—A Mixed Methods Study

**DOI:** 10.1177/23779608241305782

**Published:** 2024-12-19

**Authors:** Catherine Safari Massay, Sara Rivenes Lafontan, Jane Januarius Rogathi, Upendo S. Safari, Geofrey Nimrod Sigalla

**Affiliations:** 1 380181 Haydom Lutheran Hospital, Mbulu, Tanzania; 2 Institute of Nursing and Health Promotion, 60499 OsloMet – Oslo Metropolitan University, Oslo, Norway; 3 108094 Kilimanjaro Christian Medical University College, Moshi, Tanzania; 4 Sumve School of Nursing, Sumve, Tanzania

**Keywords:** acute illnesses, gynecology, midwifery, practice, sexually transmitted infections

## Abstract

**Introduction:**

The intrauterine device (IUD) represents the most cost-effective, long-acting reversible form of contraception, but accounts for only 1% of all contraception methods used in Tanzania.

**Objective:**

This study aims to determine the uptake of IUD use and investigate the perceptions, challenges, and recommendations surrounding the use of intrauterine devices among women of reproductive age in Tanzania.

**Method:**

A cross-sectional study was conducted including 347 women, as well as qualitative semi-structured interviews with 11 women. Quantitative data were entered into SPSS, and descriptive statistics were employed to summarize the data. Content analysis was used to analyse the qualitative data.

**Results:**

The prevalence of IUD use was 5%. The majority of the women interviewed were aware of myths and misconceptions concerning IUDs but wanted to use it after receiving information from healthcare providers.

**Conclusion:**

The study found that the use of intrauterine devices was low in the study area and that there is a need to strengthen community engagement to reduce misconceptions about the use of IUDs.

## Introduction

Reducing the unmet need for family planning is key in order to ensure sustainable development, enabling individuals and couples to make informed choices about the timing and spacing of their pregnancies (Alkema et al., 2013; Peterson et al., 2013). As defined by the World Health Organization (WHO), “women with unmet need are those who are fecund and sexually active but are not using any method of contraception, and report not wanting any more children or wanting to delay the next child” (World Health Organization, 2024). Yet, more than 200 million women of reproductive age worldwide have an unmet need for family planning. Low- and middle-income countries (LMICs) are home to a significant portion of the world's population and have the highest unmet need for family planning (Gahungu et al., 2021; Getaneh et al., 2020; Sedgh et al., 2016; Teshale, 2022; Wulifan et al., 2015). Limited access to family planning services, high levels of unintended pregnancies, and a lack of access to maternal and newborn care are all contributing factors in a majority of global maternal and newborn morbidity and mortality rates, thereby also perpetuating cycles of poverty and curtailing women's autonomy. Thus, addressing the unmet need for family planning is inextricably linked to the advancement of maternal health and broader development goals (Cottingham et al., 2012; Götmark & Andersson, 2020).

In the pursuit of meeting the unmet need for family planning, reversible long-acting contraceptives (LARCs) have emerged as valuable tools. LARCs, including intrauterine devices (IUDs) and subdermal implants, offer highly effective, long-lasting contraception. Their advantages are manifold: they require minimal user involvement, provide extended protection against unintended pregnancies, and are fully reversible upon discontinuation. Within the spectrum of contraceptive methods, IUDs, in particular, have garnered attention for their convenience, efficacy, and suitability for a diverse range of women, including those in LMICs (Friedman, 2015; Peipert et al., 2011; Stein et al., 2020; Zimmerman et al., 2021).

In LMICs, the adoption of IUDs as a contraceptive method faces a multitude of challenges that are deeply rooted in sociocultural, economic, and healthcare system factors (Muna & Mahiti, 2022). Misconceptions and myths concerning IUDs, such as concerns about their safety or their potential to cause infertility, are prevalent in many communities, which deters potential users from considering this highly effective contraceptive method (Boydell & Smith, 2023; Cohen et al., 2022). The lack of awareness and limited education on family planning options, including IUDs, further exacerbate the low uptake, as many individuals in LMICs remain unaware of the benefits and availability of these devices (Marchin et al., 2021; Sweya et al., 2016). Additionally, inadequate public awareness campaigns and the insufficient dissemination of information about contraceptive choices contribute to this issue (Bradley & Shiras, 2022; Safieh et al., 2019). The limited access to quality healthcare services, especially family planning services, presents a significant barrier to IUD utilization (Kulczycki, 2018; Muna & Mahiti, 2022).

Family planning utilization in Tanzania reflects a paradox whereby high awareness of modern contraceptive methods, at 98.1%, coexists with relatively low utilization, standing at 27% among all women (Ministry of Health, Community Development, Gender, Elderly and Children, 2016a). The unmet need for modern contraceptives is estimated at 22% (Phiri et al., 2023). These challenges are deeply entrenched in sociocultural norms and practices that are perpetuated by a patriarchal system, which restricts women's agency in crucial decisions related to reproductive choices. Recognizing these hurdles, Tanzania has made significant commitments through the Family Planning 2030 initiative. By 2025, the government aims to have increased the modern contraceptive prevalence rate (mCPR) for all women from 27% to 42% (Ministry of Health, Community Development, Gender, Elderly and Children, 2016b). With a relatively high fertility rate of 5.2 children per woman (15–49 years) and a modest 1% annual growth in the modern contraceptive rate, it is evident that sustained investments in high-impact interventions are imperative. These efforts are focused on broadening access to and utilization of family planning services, with the ultimate goal of empowering women to make informed choices about when to have children and how many children to have (Ministry of Health, Community Development, Gender, Elderly and Children, 2016b).

### Review of Literature

The current literature on family planning dynamics in Tanzania's rural areas illuminates the intricate interplay of sociocultural factors that underlie contraceptive practices within the region (Muna & Mahiti, 2022; Nkenguye et al., 2023; Sweya et al., 2016). Women's perceptions of IUDs manifest a spectrum of perspectives, which are shaped by concerns over safety and nuanced beliefs about potential side effects. Additionally, variables including access to healthcare facilities, educational level, and marital status also influence contraceptive decision-making (Abeid et al., 2023; Muna & Mahiti, 2022).

Currently, there is a significant gap in the current research, particularly within the Tanzanian context, and specifically in rural and remote areas, regarding the utilization of IUDs and the experiences of the women who use them. Research from other African countries indicates that sociocultural factors, such as traditional beliefs, misconceptions about IUD safety, and limited healthcare access, also play a critical role in influencing IUD uptake (Gahungu et al., 2021; Gueye et al., 2015; Wulifan et al., 2015; Zimmerman et al., 2021).Considering Tanzania's ongoing efforts to reduce the unmet need for family planning, there is a need for robust and up-to-date evidence in this area. This study aims to determine the uptake of IUD use and investigate the perceptions, challenges, and recommendations surrounding the use of IUDs among women of reproductive age in Tanzania.

## Methods

This study employed both qualitative and quantitative methods. A quantitative, cross-section study design was used to determine the level of uptake of IUDs. A qualitative descriptive design using semi-structured interviews was employed to gain an understanding of women's experiences about IUDs. The study was conducted from January 2018 to April 2018.

### Study Site

The study was conducted at Haydom Lutheran Hospital (HLH) in Mbulu district, in the Manyara region of Tanzania. The hospital's immediate catchment area comprises 316,000 people from four divisions in three districts—Mbulu, Hanang, and Iramba. HLH serves around 100,000 outpatients annually, and around 24,000 women attend the Reproductive- and Child Health (RCH) Clinic annually. The family planning services are provided in the hospital's RCH Clinic. In 2017, a total of 3,476 women attended the RCH Clinic for family planning services. The RCH Clinic provides almost all family planning services, including counselling and the provision of condoms, injectable contraceptives, implants, and IUDs. It is estimated that 70 women come for family planning services every week.

### Study Population and Inclusion and Exclusion Criteria

The targeted population included all women of reproductive age (between 15 and 49 years) who were seeking family planning services. The inclusion criteria were women seeking family planning services at the RCH Clinic and who had provided signed, informed consent to participate in the study. The exclusion criteria were all women with gynaecological morbidities, such as cervical cancer and pelvic inflammatory diseases.

### Sample Size Determination

The sample size was calculated by considering a 95% confidence level and a margin of error of 3%, while assuming a population proportion of 7.9%, where *n* = required sample size, *p* = estimated population proportion, and *ε* = margin of error:

$$n=\frac{z^{2}pq}{\varepsilon^{2}}$$

where,

*n* = Minimum sample size
*p* = Proportion of women using IUD = 7.9% (Mbuthia, 2017).
*q* = Probability of women who ever use IUD, 1−p=92.1%
*ε* = Marginal error = 3%

Therefore,

$$n=\frac{1.96^{2}*0.079*0.921}{0.03^{2}}=311$$

By adding a 10% non-response rate (311 + 31 = 342), the final sample size was 342 participants.

An average of 290 women attend the RCH Clinic at HLH Hospital monthly. Over a data collection period of 3 months, it was estimated that 870 women could be expected to attend the regular RCH Clinic.

Selecting a sample of 342, thus K=(N/n)=870÷342=3 .

Every third person was selected to represent the participants in the study. The first participant was selected using a simple random sampling technique employing the ballot method.

### Sampling Technique

A systematic sampling technique was employed to obtain the study participants. The women's attendance was recorded on a paper list on each day of the data collection. One of the members of staff received training and instruction as to how to use the list provided; she/he therefore had sole responsibility for directing the clients to the respective desk for interview.

### Quantitative Data Collection

A structured questionnaire in Swahili was used for the qualitative data collection. Data collectors were trained over a period of one day before data collection started. To recruit participants, the research team introduced themselves to the women and asked for their consent to participate in the study. To ensure that women with limited literacy skills were also included in the study, research assistants were employed to assist participants in completing the questionnaire. To ensure privacy, a separate room was used to interview the participants using the questionnaire. No one except the research assistant, the participant, or a child less than two years of age was allowed to be present during the interview.

### Qualitative Data Collection

Semi-structured interviews were conducted with 11 women who were using an IUD with the aim of identifying their experiences of using the device. All interviews were conducted in Kiswahili by the first author, who is a Tanzanian midwife familiar with the study context. An interview guide was used to collect qualitative data regarding users' experiences of IUDs, with questions such as “What information were you given regarding IUDs?,” and “Now you have been using the IUD, what is your experience with this method?” Only women who had used an IUD for more than 6 weeks were invited to participate. A tape recorder was used to record each interview, and the interviews lasted for 20 to 30 minutes. Reflexive notes were written after each interview.

### Quantitative Data Management and Analysis

The quantitative data were entered into Statistical Package for the Social Scientist (SPSS Version 23.0). Descriptive statistics were employed to summarize the data, whereby mean and standard deviation were used to summarize non-categorical data that were normally distributed; otherwise, median, and range values were used instead. Frequency and percentages were employed to summarize categorical data (age group, level of education, types of contraceptives, level of occupation, and parity group).

### Qualitative Data Analysis

After data collection, all recorded interviews were transcribed verbatim and translated into English. The transcripts were analyzed for thematic content using an inductive approach. After familiarization with the dataset by reading and re-reading the text, the data were searched for repeated patterns of meaning relevant to the areas of interest. An initial set of descriptive codes was generated by the first author. After deciding on a final set of codes, the codes were grouped into themes. All authors revised and agreed on the final set of codes and themes.

### Ethical Considerations

Participants were informed of the full nature of the study, after which they provided signed, informed consent to participate in the study. Consent for those who were below 18 years of age was countersigned by a guardian. Privacy and confidentiality were maintained during interviews, and participant information was de-identified using participant ID. Participation in the study was voluntary, and participants were informed of their right to withdraw from the study without any consequences.

The ethical approval to conduct this study was received from Kilimanjaro Christian Medical College Research Ethics and Review Committee (CRERC) with certificate number 1098. Permission was also obtained from the medical Director of Haydom Lutheran Hospital, who informed the Head of Department of RCH clinic about the study.

## Results

### Sociodemographic Characteristics of the Participants

There were a total of 347 participants, and the response rate was 100%. The mean age of the 347 participants interviewed was 28.6 years, with a standard deviation (SD) of 6.5 years (Table 1). Their age ranged from 16 to 49 years. The majority of participants (65.7%) had completed primary education and 85.3% were married. More than two thirds of participants (81.3%) were farmers. Around three-fifths of participants' partners (61.1%) had completed primary education, and more than two-thirds of them (67.1%) were farmers. The median parity was three (range 1–12) births. Two-thirds (62.8%) reported having between two and five living children. The average family size was five people and ranged from 1 to 16, and the majority of participants (62.5%) lived in a household that had more than five people.

**Table 1.** Sociodemographic Characteristics of the Participants (*n* = 347).

Variables	Number (*n*)	Percentage (%)
Age (years)		
16–24	101	29.1
25–34	184	53.0
≥35	62	17.9
Education level		
No formal education	28	8.1
Primary education	228	65.7
Secondary education	84	24.2
Tertiary education (college/university)	7	2.0
Marital status		
Never married	44	12.7
Married/cohabiting	296	85.3
Divorced/separated/widowed	7	2.0
Occupation		
Employed	13	3.7
Business owner	38	11.0
Farmer	282	81.3
Student	14	4.0
Parity		
1	65	18.7
2–5	221	63.7
6 and above	61	17.6

### Prevalence of Uptake of Modern Family Planning Methods

**Figure 1.**
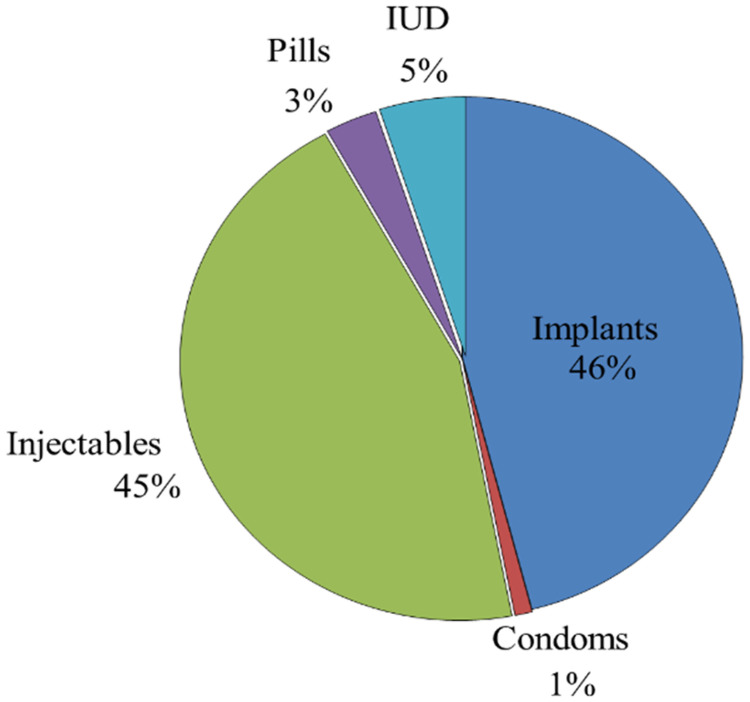
The prevalence of uptake of modern family planning methods among women attending the reproductive and child health clinic at HLH hospital (*n* = 347).

The prevalence of uptake of modern family planning methods is illustrated in Figure 1. Implants were the most preferred method of family planning (46% of participants), followed by injectable contraceptives (45%). About 5% of all participants reported using an IUD as their family planning method. No one opted for bilateral tubal ligation.

### Source of Information About IUDs

More than three quarters of participants (275, 79.3%) reported that they had heard of IUDs before visiting a health facility on the day of interview. Of those who had previously heard of IUDs, 164 (59.6%) had received information about IUDs from healthcare providers, 56 (20.4%) from friends/relatives, 4 (1.5%) from mass media, 1 (0.4%) at school, and 36 (13.1%) had received information from multiple sources. However, 14 (5.1%) participants did not remember the exact source of the information.

### Qualitative Results

To understand the participants' experiences of IUDs, eleven participants who were using an IUD were interviewed, and their characteristics are presented in Table 2, below. In general, the interviewed participants were aged between 20 and 45 years, and had between one and nine children. These participants had been using an IUD for a period of between 2 months and 2 years, with an average duration of use of 1 year. The majority of these women (8) were educated to primary level or below.

**Table 2.** Characteristics of Women Participating in the Qualitative Interview (*n* = 11).

Age (years)	Number of children	Duration of IUD use	Educational level
42	4	6 months	Secondary education
29	3	2 months	Primary education
45	8	2 years	Primary education
45	8	1 year	No formal education
32	2	2 years	Secondary education
41	9	1 year	No formal education
20	1	4 months	Primary education
38	6	2 months	No formal education
43	5	3 months	No formal education
36	4	3 months	Primary education
28	3	2 years	Secondary education

Table 3 summarize the themes and key findings below.

**Table 3.** Themes and Key Findings.

Theme	Details
Advantages of Using IUD	Long-acting family planning method suitable for women wanting to defer childbirth for over three years (8 out of 11 participants). No need for frequent healthcare visits unless side effects are experienced. Immediate effectiveness after insertion; no need for additional contraceptive methods (e.g., condoms). Minimal side effects compared to other hormonal methods. Fertility quickly returns after removal.
Challenges Using IUD	Discomfort with insertion, especially the notion of something being inserted via the private part. Side effects: abnormal vaginal bleeding, discharge, abdominal pain during menstruation, and pelvic cramping. Side effects were usually experienced during the early use period and ranged from minor to moderately severe.
Myths Surrounding IUD Use	Fears in the community about the IUD moving from the uterus to other parts of the body. Beliefs that IUD could cause cancer or be pushed away during sexual activity.
Recommendations for Increasing IUD Use	Providing correct information about IUDs empowers women to make informed decisions. Health education from care providers (especially nurses) is essential to combat negative perceptions and myths. Counseling needs to be more detailed to ensure women fully understand the method. Training healthcare providers on IUD insertion is necessary as some facilities lack adequate expertise.

### Advantages of Using IUD

Most of the participants interviewed (8 of 11) interviewed reported the use of IUDs as being a long-acting family planning method that was suitable for them. This was especially the case for women who desired to defer having children for a period of more than three years. The use of an IUD did not require the user to frequently visit a healthcare facility, unless they experienced side effects. An additional advantage of this method was that it was immediately effective, and did not require the use of another family planning method:

*I received information from the nurse that this method is good and starts working immediately after insertion—there is no need to wait for some days for it to be effective. Therefore, there is no need to abstain from sexual intercourse or use a condom, as there is no delay in its action. (P_5_)*

Comparative to other methods, IUDs were specifically described by most participants as having minimal side effects. This was also highlighted by participants who had previously used other hormonal methods. Another advantage of the method raised by the participants was that fertility returned quickly after removal for those who planned to conceive in the future:

*I am very happy because I am doing my activities without problem. When I wanted to conceive, could just remove it and conceive again.* (P_10_)

### Challenges of Using an IUD

Despite the positive experiences described by the majority of the participants, several had also experienced challenges, which were primarily related to the insertion of the device or its side effects:

*It is not easy for someone to accept something that is inserted into their private parts. It was not easy for me to take such a decision, and I felt very shameful and afraid of pain during the insertion.* (P_1_)

Participants spelt out four main unwanted experiences of side effects that result from the use of IUDs: abnormal vaginal bleeding, vaginal discharge, abdominal pain during menstruation, and pelvic cramping. However, three participants who recounted their experiences of side effects described them as having happened during the early period of use and varying from minor to moderate severity:

*At the beginning, I experienced pelvic cramping as a pain below the umbilicus, which was accompanied by some vaginal discharge, but the condition subsided after a short period. Later, I had heavier menstrual bleeding, especially during the first three to six months of use, but after that, no such experiences. I am now happy with the method.* (P_4_)

### Myths Surrounding IUD Use

The majority of women who were using IUDs described fears associated with the use of these devices. Several stated that, in the community in which they lived, IUDs were believed to move from the uterus to other parts of the body. The movement of the IUD to other areas in the body was believed to take place during the sexual act or when it failed to work. Describing these beliefs, one participant who had used the method for a year reported that:

*Many women are afraid to use an IUD and believe that it can cause cancer, can be pushed away by their partner during sex, and it can get lost inside.* (P_4_)

The sharing of experiences by other women who had been using the method increased their confidence to refute these beliefs and enhanced the acceptance of IUD use. It was also noted that none of the participants had ever experienced the IUD disappearing from the uterus or being discovered at another site in their body.

### Recommendations About How IUD Use Can Be Increased

Most of the participants believed that IUDs were the best family planning method because they had received initial information about IUDs from health care providers, and they were recommended by other women. They believed that having correct information about IUDs empowers women to make informed decisions about their method of choice. Explaining how she opted for the method, a 36-year-old participant with four children who would like to have another two children in the future said:

*I was taught about this method by a nurse when I was asking for advice on the best family planning, and she told me this is one of the best family planning methods which can stay in for as long you would like. I haven’t had any problems since I’ve been using it, and I have had the intrauterine device for three months now. Even my period is regular and I’ve had no problems with my health since I started using this method.* (P_10_)

Most participants believed that, due to the negative perceptions in the community about IUDs, healthcare providers needed to devote a lot of time to providing counselling. One participant recommended the following:

*The information about intrauterine devices is inadequate. I think there is a need for health education for mothers. Healthcare providers—especially nurses—should have the chance to talk with mothers about this method because I’m sure that many mothers are not aware of it. Personally, I think the information was insufficient and inadequate—I think I missed a lot of the information I needed before opting for the method.* (P_2_)

Two participants recommended improving healthcare providers' competence for inserting IUDs as they reported that several health facilities provided information about the method but lacked adequate knowledge about how to insert the device in those women who wanted it.

## Discussion

The study findings indicate a relatively low uptake of IUDs, with only 5% of participants reporting the use of IUDs as their preferred family planning method. This aligns with the broader trend in LMICs, where various studies have reported relatively low utilization of IUDs compared to other contraceptive methods (Abeid et al., 2023; Alkema et al., 2013). Factors such as sociocultural norms and misconceptions about IUDs can hinder their adoption (Abeid et al., 2023; Peipert et al., 2011). The study findings mirrored these challenges, as some participants expressed fears and the prevalence of myths related to IUD use, thereby reinforcing the importance of addressing these misconceptions to increase uptake.

Despite the high awareness of contraceptives in Tanzania, there is a low level of utilization. This paradox can be attributed to the sociocultural norms and misconceptions surrounding contraceptive methods, such as IUDs, which are prevalent across the sub-Saharan region (Gahungu et al., 2021; Gueye et al., 2015; Sedgh et al., 2016). Cultural beliefs that value large families for social and economic reasons often discourage the use of long-term contraceptives such as IUDs (Abeid et al., 2023; Alkema et al., 2013). Additionally, widespread misconceptions about the health risks and complications associated with IUDs, along with religious influences and gender dynamics limiting women's autonomy, further hinder their adoption (Gueye et al., 2015; Sedgh et al., 2016; Stats et al., 2020). These factors may partly explain why the IUD use was so low in the current study. In LMICs, health services in resource-limited settings often lack the necessary infrastructure and resources to offer IUD insertion and follow-up services, which restricts women's access to this contraceptive method (Abeid et al., 2023). Cultural norms and societal pressure can also play a role in discouraging the use of IUDs (Gahungu et al., 2021; Gueye et al., 2015; Sedgh et al., 2016). The qualitative findings from the present study found that misconceptions about IUDs were prevalent in the communities, and this may be a contributing factor to the low uptake. In some LMICs, cultural beliefs and social expectations surrounding fertility and family planning can discourage women from considering IUDs as an option (Muna & Mahiti, 2022). Stigmas associated with contraceptive use can deter individuals from seeking out family planning services, including the provision of IUDs (Boydell & Smith, 2023). Economic factors, such as the cost of IUDs and the expenses related to insertion and follow-up appointments, may present additional barriers (Muna & Mahiti, 2022). Affordability remains a considerable concern for many individuals in LMICs, and the financial burden associated with IUD use can be a deterrent (Rwegoshora et al., 2020). Although family planning services are free in the clinic used by the participants included in the present study, there are costs associated with accessing the services, such as expenses related to travel. The costs related to accessing care, including costs related to transportation to the health facility, have been found to be a barrier preventing access to family planning services for women in both high- and low-income countries (Amissah et al., 2020; Bouanchaud et al., 2022; Fuentes et al., 2023). Factors related to the healthcare system itself may also present barriers to access, such as the absence of consistent and effective supply chain management systems, which may lead to stockouts of IUDs (Nkenguye et al., 2023). Other barriers include a shortage of trained healthcare providers capable of offering IUD services, and a lack of available facilities that are equipped for safe IUD insertion (Abeid et al., 2023). Ethical concerns and the potential risk of contraceptive coercion remain important issues in family planning, highlighting the need for strict ethical guidelines when training healthcare providers in family planning counselling, including IUD counselling (Boydell & Smith, 2023; Montt-Maray et al., 2023).

In the qualitative interviews, participants highlighted the advantages of IUDs, including their long-acting nature, minimal side effects, and rapid fertility return after removal, which is essential for women who plan to conceive in the future. These advantages were consistent with findings from other studies from LMICs, which also identified these aspects as factors that could positively influence IUD utilization (Rwegoshora et al., 2020; Sweya et al., 2016). The comparatively low occurrence of side effects of IUDs, as reported by the participants in the present study, is particularly important when promoting the method, as there is a higher acceptability of contraceptive methods with fewer side effects (Rwegoshora et al., 2020; Sweya et al., 2016).

The study findings highlight the need for skilled health providers with training in IUD counselling and insertion. Counselling for contraceptive users and potential users is widely accepted as a strategy to increase acceptance, adherence, continuation, and user satisfaction (Modesto et al., 2014). It has also been found to reduce premature discontinuations due to side effects. With the use of LARC, premature discontinuation can reduce its effectiveness and potentially lead to unplanned pregnancies (Modesto et al., 2014). This shows the importance of counselling being an ongoing activity during contraceptive use to address side effects and reinforce the benefits of the chosen method, thus reducing premature discontinuation (Cavallaro et al., 2020). It is also worth noting that interventions aimed at providing counselling for women outside of family planning services have been linked with increased contraceptive use, especially in contexts with expanded contraceptive provision (Rwegoshora et al., 2020). As there was a high level of reported misconceptions about IUDs in the communities where the study was conducted, the use of community outreach activities to increase awareness about family planning methods could be one mitigating effort to tackle this issue (Cavallaro et al., 2020). Furthermore, additional counselling sessions during pregnancy or postpartum appear to enhance postpartum contraceptive use, including IUDs, regardless of their timing (Cavallaro et al., 2020; Fatima et al., 2018; Karra et al., 2017; Ndegwa et al., 2014).

### Strengths and Limitations of the Study

The present study provides important new knowledge about the use and perceptions of IUDs among women in northern Tanzania, where many of the participants live in remote and rural areas. A strength of this study is that lessons learned from this study can inform similar initiatives in other low-resource settings that face comparable challenges, thereby contributing to global efforts to enhance family planning services and reproductive health outcomes. The present study has explored the participants' views on the uptake of IUDs and did not interview healthcare providers, who could have provided important views on the study subject. It would also have been interesting to include women who chose to not use an IUD to find out more about their perceptions. This was a hospital-based study and the results have limited generalization to other regions or healthcare settings in Tanzania. The first author who conducted the study had limited research experience, which was compensated for by the research experience of the co-authors. To minimize the influence of biases during data collection, the researchers practiced reflexivity, regularly reflecting on their beliefs and assumptions to ensure they remained aware of potential biases. The interviewers received thorough training to maintain neutrality and consistency in questioning, using a structured and neutral interview guide. Peer debriefing with colleagues was also used to identify any unintentional biases and data triangulation to ensure richness of the data. These strategies helped to ensure that the data collection process remained objective, and that participants' voices were authentically represented.

### Implications for Practice

Nurses are at the forefront of reproductive health education and counselling, which makes it essential for them to understand and address the factors affecting IUD uptake. They can effectively educate women who are seeking family planning services, empowering them to make informed decisions about their contraceptive options. By incorporating evidence-based information into counselling sessions, focusing on the benefits of IUDs can empower women to make informed decisions about their contraceptive options. This may also include offering practical demonstrations of IUD insertion procedures or using visual aids. In order to increase the uptake of long-acting contraceptive methods such as IUDs, it is crucial for nurses and other healthcare providers to adopt culturally sensitive care approaches. To further support this, healthcare providers can implement training programs or workshops that focus on IUD counselling and insertion techniques. These could be integrated into continuous professional development initiatives, ensuring that providers are well-equipped to deliver high-quality care. By actively engaging with community leaders and utilizing local languages in their counselling sessions, they can ensure that information is accessible and culturally relevant. Furthermore, organizing community workshops that involve both healthcare providers and community members could enhance the understanding and acceptance of IUDs. Nurses can also collaborate with policymakers to advocate for the availability of affordable IUDs in health facilities and for the continuous training of healthcare providers to ensure high-quality family planning services.

## Conclusions

The study revealed a low uptake of IUDs, with only 5% of participants in the study setting choosing these as their contraceptive method. The low uptake was influenced by sociocultural norms, misconceptions, and concerns about IUD-related side effects. To address these barriers and promote IUD use, the study highlights the need for skilled healthcare providers and effective counselling. Additionally, community outreach can also play a crucial role in dispelling misconceptions and encouraging postpartum contraceptive use, including IUDs. The implementation of these strategies would be in alignment with Tanzania's overarching goal of reducing the unmet need for family planning, thereby contributing to improved maternal and child health, and overall socioeconomic development in the country.
